# Supplementary material for: Household transmission of the SARS-CoV-2 Omicron variant in Denmark
Source: Nat Commun. 2022 Sep 23;13:5573. doi: 10.1038/s41467-022-33328-3 (PMC9508106; doi:10.1038/s41467-022-33328-3)
Supplement: Supplementary file 1 — Supplementary Information [file 41467_2022_33328_MOESM1_ESM.pdf]

Supplementary Information

Household transmission of the SARS-CoV-2 Omicron variant in Denmark

Frederik Plesner Lyngse[1,2,3,*], Laust Hvas Mortensen[4,5],

Matthew J. Denwood[6], Lasse Engbo Christiansen[7],

Camilla Holten Møller[3], Robert Leo Skov[3],

Katja Spiess[3], Anders Fomsgaard[3],

Maria Magdalena Lassaunière[3], Morten Rasmussen[3],

Marc Stegger[8], Claus Nielsen[3],

Raphael Niklaus Sieber[8], Arieh Sierra Cohen[3],

Frederik Trier Møller[3], Maria Overvad[3],

Kåre Mølbak[3], Tyra Grove Krause[3],

Carsten Thure Kirkeby[6]

[*] Correspondence to Frederik Plesner Lyngse, fpl@econ.ku.dk. Affiliations: [1]Department of Economics & Center for Economic Behavior and Inequality, University of Copenhagen, Øster Farimagsgade 5, DK-1353 Copenhagen K, Denmark; [2]Danish Ministry of Health, Holbergsgade 6, DK-1057, Copenhagen K, Denmark; [3]Statens Serum Institut, Artillerivej 5, DK-2300 Copenhagen S, Denmark; [4]Statistics Denmark, Sejrøgade 11, DK-2100 Copenhagen; [5]Department of Public Health, Faculty of Health and Medical Sciences, University of Copenhagen, Øster Farimagsgade 5, DK-1353 Copenhagen K, Denmark; [6]Department of Veterinary and Animal Sciences, Faculty of Health and Medical Sciences, University of Copenhagen, Grønnegårdsvej 8, DK-1870 Frederiksberg C, Copenhagen, Denmark. [7]Department of Applied Mathematics and Computer Science, Dynamical Systems, Technical University of Denmark, Richard Petersens Plads, 324, DK-2800 Kgs. Lyngby, Denmark.; [8]Department of Bacteria, Parasites and Fungi, Statens Serum Institut, Artillerivej 5, DK-2300 Copenhagen S, Denmark.

1 Background

This section provides some background information on the circumstances surrounding our study, i.e., the situation in Denmark between 1st-31st December 2021. Table S1 shows the number of cases identified with RT-PCR, the proportion of positive cases selected for Variant PCR testing and the proportion of positive cases selected for Variant PCR for which Omicron was detected.

Table S1: Daily number of cases detected in Denmark during December 2021

Sample date		Positive RT-PCR tests (N)	Selected for Variant PCR (%)	With Omicron (%)
01DEC21	Wednesday	4,563	96	2
02DEC21	Thursday	4,606	94	1
03DEC21	Friday	5,212	96	2
04DEC21	Saturday	5,132	97	2
05DEC21	Sunday	4,827	97	4
06DEC21	Monday	7,131	96	5
07DEC21	Tuesday	7,380	96	8
08DEC21	Wednesday	6,729	95	11
09DEC21	Thursday	6,678	95	12
10DEC21	Friday	6,974	94	13
11DEC21	Saturday	6,717	95	17
12DEC21	Sunday	7,181	93	23
13DEC21	Monday	10,642	90	30
14DEC21	Tuesday	11,566	94	41
15DEC21	Wednesday	11,264	94	48
16DEC21	Thursday	10,610	93	48
17DEC21	Friday	11,089	92	54
18DEC21	Saturday	10,486	86	57
19DEC21	Sunday	10,650	72	63
20DEC21	Monday	13,950	20	63
21DEC21	Tuesday	13,726	24	76
22DEC21	Wednesday	12,317	11	76
23DEC21	Thursday	13,300	26	78
24DEC21	Friday	7,434	9	78
25DEC21	Saturday	8,296	12	77
26DEC21	Sunday	11,581	20	85
27DEC21	Monday	24,029	20	88
28DEC21	Tuesday	23,341	7	87
29DEC21	Wednesday	18,401	3	85
30DEC21	Thursday	20,477	5	86
31DEC21	Friday	10,299	5	85

Notes: This table provides number of positive RT-PCR tests along with the proportion of those selected for Variant PCR and the proportion of those identified with Omicron (relative to Delta) for December 2021. See appendix Tables S2 and S3 for the number of antigen and RT-PCR tests performed.

1.1 Time to test result

During the study period, testing with both antigen and RT-PCR tests was available to all residents of Denmark free of charge. Antigen tests provide a quick test result (positive/negative) with a median time from sample to result of less than 30 minutes (Table S2). All positive antigen tests were recommended by the authorities to be confirmed with an RT-PCR test. RT-PCR tests are more sensitive,[1] but also require a longer time before the result is known. The median time to known result is approximately 24 hours (Table S3). Only samples with positive RT-PCR test results were available for selection for Variant PCR and whole genome sequencing (WGS).

Table S2: Time between sampling and test results for antigen tests (minutes)

Sample date		Time to test result, minutes P5	Q1	Median	Q3	P95	Number of tests
01DEC21	Wednesday	8	16	20	25	35	188,052
02DEC21	Thursday	9	17	22	29	42	216,548
03DEC21	Friday	9	18	24	30	35	189,593
04DEC21	Saturday	12	20	23	28	33	142,053
05DEC21	Sunday	11	16	19	24	29	149,098
06DEC21	Monday	8	13	19	26	32	211,712
07DEC21	Tuesday	9	15	22	26	31	211,038
08DEC21	Wednesday	9	15	20	25	29	207,141
09DEC21	Thursday	10	16	22	27	33	245,058
10DEC21	Friday	9	19	25	30	37	213,063
11DEC21	Saturday	13	20	24	30	35	155,858
12DEC21	Sunday	11	16	21	26	32	167,670
13DEC21	Monday	8	16	22	27	36	232,668
14DEC21	Tuesday	9	20	25	30	38	225,263
15DEC21	Wednesday	8	21	26	32	41	219,478
16DEC21	Thursday	10	22	29	35	45	258,491
17DEC21	Friday	10	29	34	39	47	236,644
18DEC21	Saturday	17	29	34	38	47	176,424
19DEC21	Sunday	12	28	31	33	39	182,974
20DEC21	Monday	10	25	29	35	45	271,191
21DEC21	Tuesday	11	26	32	40	48	258,469
22DEC21	Wednesday	10	26	33	41	48	272,965
23DEC21	Thursday	10	32	38	47	56	246,286
24DEC21	Friday	8	32	34	36	41	72,578
25DEC21	Saturday	7	17	20	22	30	73,516
26DEC21	Sunday	7	11	12	15	31	82,131
27DEC21	Monday	8	12	16	21	28	186,664
28DEC21	Tuesday	8	13	17	19	27	195,094
29DEC21	Wednesday	8	13	16	21	28	216,572
30DEC21	Thursday	8	13	18	23	30	229,325
31DEC21	Friday	7	11	13	15	35	72,615

Notes: This table provides summary statistics on the time from sampling to the test result (positive/negative). P5 = 5th percentile, Q1 = 1st quartile, Q3 = 3rd quartile, P95 = 95th percentile.

Table S3: Time between sampling and test results for RT-PCR tests (hours)

Sample date		P5	Q1	Median	Q3	P95	Number of PCR tests
		Time to test result, hours					
01DEC21	Wednesday	16	20	22	26	36	177,466
02DEC21	Thursday	16	20	22	27	41	217,979
03DEC21	Friday	16	20	22	28	42	237,466
04DEC21	Saturday	16	20	24	28	40	143,557
05DEC21	Sunday	16	20	23	27	37	144,767
06DEC21	Monday	16	20	22	27	39	228,352
07DEC21	Tuesday	16	20	22	27	39	233,073
08DEC21	Wednesday	16	20	23	28	41	239,456
09DEC21	Thursday	16	20	22	28	41	268,387
10DEC21	Friday	16	20	23	28	41	274,849
11DEC21	Saturday	16	20	24	29	40	178,176
12DEC21	Sunday	16	20	24	28	37	178,068
13DEC21	Monday	16	20	23	28	41	261,261
14DEC21	Tuesday	16	20	22	27	40	254,258
15DEC21	Wednesday	16	20	24	29	42	225,026
16DEC21	Thursday	16	20	24	29	42	255,303
17DEC21	Friday	16	20	24	29	45	273,106
18DEC21	Saturday	16	21	25	29	42	221,579
19DEC21	Sunday	16	20	24	29	39	220,878
20DEC21	Monday	16	20	24	28	41	248,232
21DEC21	Tuesday	16	20	24	28	39	251,443
22DEC21	Wednesday	16	20	24	29	40	275,255
23DEC21	Thursday	16	20	24	30	85	277,456
24DEC21	Friday	16	20	24	28	38	155,248
25DEC21	Saturday	16	19	23	27	36	128,703
26DEC21	Sunday	16	20	23	27	36	168,399
27DEC21	Monday	16	19	23	27	35	207,265
28DEC21	Tuesday	16	19	23	27	36	213,340
29DEC21	Wednesday	16	20	23	27	36	238,567
30DEC21	Thursday	16	20	24	29	39	314,614
31DEC21	Friday	16	20	23	27	35	181,564

Notes: This table provides summary statistics on the time from sampling to the test result (positive/negative). P5 = 5th percentile, Q1 = 1st quartile, Q3 = 3rd quartile, P95 = 95th percentile. See appendix Table S1 for the number of positive RT-PCR tests, including the proportion identified as Omicron.

1.2 Probability of sampling for Variant PCR

This subsection provides details on the sampling probability for variant PCR during the time interval corresponding to the inclusion period for primary cases (9th-15th December 2021). Figure S1 shows the proportion of positive RT-PCR samples selected for Variant PCR testing as well as the proportion of positive samples with the Omicron VOC. The probability that a positive RT-PCR sample was selected for Variant PCR testing (purple) was extremely high, with no evidence for selection bias depending on either sample Ct value (panel a) or age (panel b). However, a higher proportion of cases aged 15-30 years tested positive with the Omicron VOC relative to other age groups (green dashed line, panel b). This confirms that household characteristics are confounded with variant.

Figure S1: Probability of sampling for Variant PCR

(a) Ct value (b) Age

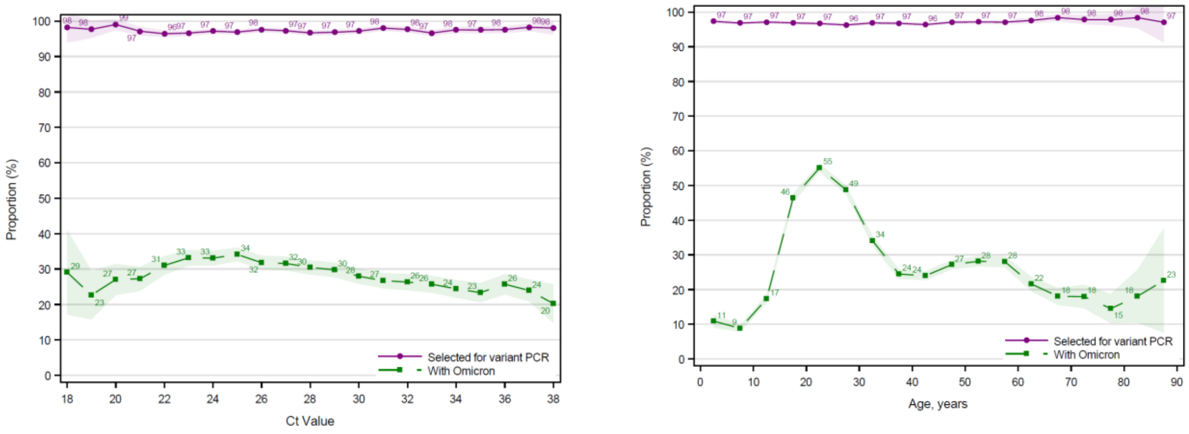

Notes: This figure shows the proportion of positive RT-PCR samples selected (purple) for Variant PCR testing and the proportion testing positive (green) with the Omicron VOC. Panel (a) shows the selection by Ct value; panel (b) by age. Only positive RT-PCR tests from 9th-15th December 2021 performed by TestCenter Denmark are included. The markers show the point estimates of the mean. The shaded areas show the 95% confidence bands.

1.3 Robustness of Variant PCR results

This subsection provides additional results of the validity of the Variant PCR, i.e., the laboratory test used to distinguish between Omicron and Delta in positive RT-PCR tests, using high quality whole-genome sequencing (WGS).

Of the 326,588 positive RT-PCR tests between 1st-31st December 2021, 39,683 had a Variant PCR result and a successfully sequenced genome (Table S4). Of these samples, the Variant PCR test categorized 10,637 as Omicron and 29,046 as "not Omicron". The WGS results showed that 35 (0.33%) of the Omicron classifications were incorrect, and that 257 (0.88%) of the "not Omicron" classifications were incorrect (Table S5). This extremely high agreement confirms that our study is not affected by any classification bias with respect to variant.

Table S4 Sample selection for validation of PCR test using whole genome sequencing (WGS)

	Number
RT-PCR positive samples	326,588
Selected for Variant PCR	160,767
Selected for WGS	56,646
Successful WGS	44,669
Variant PCR & WGS result	39,683

Notes: This table shows the number of positive RT-PCR samples and the number selected for Variant PCR and WGS as well as having a successfully sequenced genome. The samples were taken from 1-31 December 2021.

Table S5 Validation of Variant PCR test using whole genome sequencing (WGS)

	WGS			False positive	False negative
Variant PCR	Omicron	Not Omicron	Total	rate (%)	rate (%)
Omicron	10,602	35	10,637	0.33	
Not Omicron	257	28,789	29,046		0.88
Total	10,859	28,824	39,683		

Notes: This table shows the number of positive RT-PCR samples with both a Variant PCR test result and a successfully sequenced genome (see appendix Table S4 for selection of sample). The Variant PCR test identified 10,637 as Omicron and 29,046 as "not Omicron". The WGS results showed that 35 of the 10,637 samples identified by the Variant PCR as Omicron were in fact "not Omicron", implying a false positive rate for Omicron of 0.33% (35/10,637). Similarly, the WGS results showed that 257 of the 29,046 samples identified by the Variant PCR as "not Omicron" were in fact Omicron, implying a false negative rate for Omicron of 0.88% (257/29,046).

2 Causal assumptions

The causal effect of household exposure to the Omicron VOC rather than the Delta VOC on the SAR may be confounded. We assume that these differences are caused by the temporo-spatial patterns of transmission of the Omicron VOC when first introduced in Denmark (see appendix Figure S2). The directed acyclic graph displayed in Figure S2 suggests a confounding pathway from variant to SAR via the initial chains of transmission through household characteristics. What the graph encodes is that we believe that any differences between households exposed to the Omicron VOC and those exposed to the Delta VOC are due to the particularities of how Omicron was initially spread throughout the community. While the Delta VOC was widespread at the start of the study period, the Omicron VOC was not widespread. The household structure and other characteristics of the households exposed to the two variants might therefore differ. A causal interpretation of our findings is conditional on the assumption that all effects of the non-random assignment of variants to households are intercepted by conditioning on the observed household characteristics. We note that this will also diminish any household unobserved characteristics that are associated with the observed characteristics, e.g., age-related behavioral factors will be indirectly adjusted through the adjustment for age.

Figure S2: Directed acyclic graph (DAG) showing the causal inference of the study.

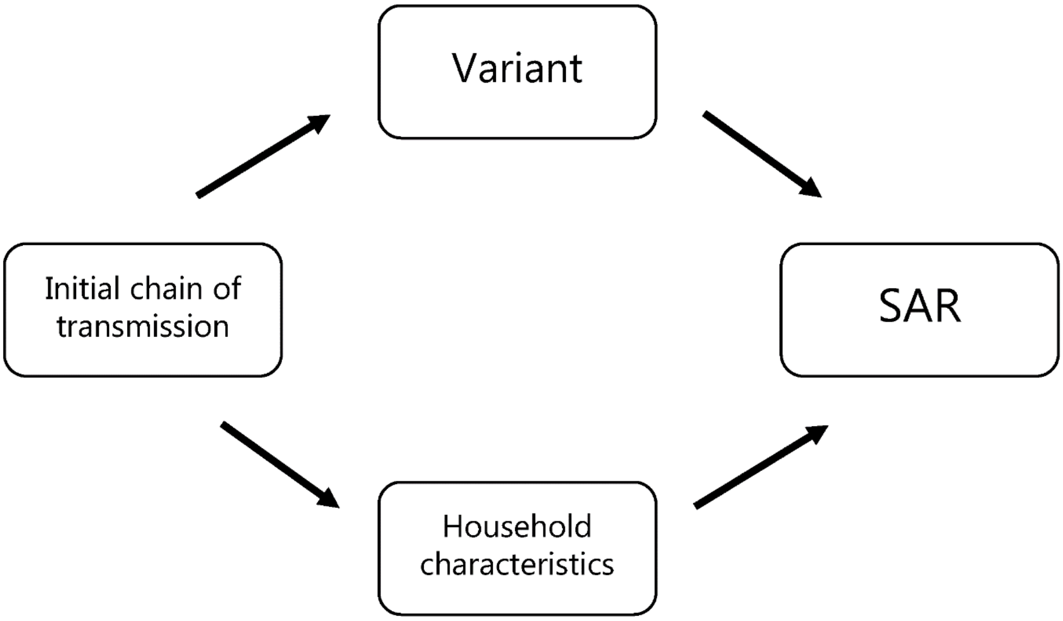

Notes: The initial chain of transmission affects the estimated SAR through both the household characteristics and the variant within the household.

3 Descriptive statistics

In this section, we provide additional descriptive statistics on our study sample.

Appendix Table S6 shows the summary statistics at the level of primary case (this augments Table 1, which shows the summary statistics for primary cases and contacts separately).

Appendix Tables S7 and S8 show more detailed summary statistics on the vaccination status of individuals within the “Fully vaccinated” category.

Appendix Table S9 shows the SAR and number of observations by vaccination status of both the primary case and contact.

Appendix Table S10 shows SAR and number of observations by vaccination status and age of the primary case.

Appendix Table S11 shows SAR and number of observations by vaccination status and age of the contact.

Appendix Table S12 shows SAR and number of observations by household size and vaccination status of the contact.

Table S6: Summary statistics by primary case level

	Omicron				Delta			
	Primary Cases	Household Contacts	Secondary Cases	SAR (%)	Primary Cases	Household Contacts	Secondary Cases	SAR (%)
**Total**	8,568	18,038	5,229	29	18,107	42,964	8,911	21
**Sex**								
Male	4,417	9,393	2,835	30	9,257	21,971	4,668	21
Female	4,151	8,645	2,394	28	8,850	20,993	4,243	20
**Age**								
0-10 years	417	1,228	443	36	4,475	13,351	2,727	20
10-20 years	1,875	4,883	954	20	3,507	9,688	1,482	15
20-30 years	2,755	4,596	1,096	24	2,432	4,358	677	16
30-40 years	1,186	2,665	866	32	1,909	4,747	1,230	26
40-50 years	1,094	2,731	1,039	38	2,312	5,729	1,362	24
50-60 years	874	1,505	618	41	2,056	3,428	895	26
60-70 years	280	334	167	50	1,019	1,230	407	33
70+ years	87	96	46	48	397	433	131	30
**Household size**								
2 persons	3,339	3,339	1,266	38	5,564	5,564	1,584	28
3 persons	2,102	4,204	1,179	28	3,863	7,726	1,552	20
4 persons	2,190	6,570	1,894	29	5,632	16,896	3,451	20
5 persons	760	3,040	734	24	2,462	9,848	1,884	19
6 persons	177	885	156	18	586	2,930	440	15
**Immunity**								
Unvaccinated	1,166	2,936	904	31	8,611	23,694	5,086	21
Fully vaccinated	6,934	14,318	4,076	28	8,968	18,461	3,679	20
Booster vaccinated	468	784	249	32	528	809	146	18

Notes: This table shows the household contacts, secondary cases and SAR for both Omicron and Delta, defined by the primary case. For instance, there were 417 primary cases with Omicron aged 0-10 years, which had 1,228 household contacts of which 443 tested positive, implying a SAR of 36% from 0 cases with Omicron aged 0-10 years.

Table S7: Summary statistics of vaccination status

	Omicron				Delta			
	Primary Cases	Household Contacts	Secondary Cases	SAR (%)	Primary Cases	Household Contacts	Secondary Cases	SAR (%)
**Total**	8,568	18,038	5,229	29	18,107	42,964	8,911	21
**Immunity**								
Unvaccinated	1,166	4,171	1,155	28	8,611	13,750	3,718	27
Previous infection (no vaccination)	128	479	70	15	145	920	49	5
Fully vaccinated (no previous infection)	6,392	11,124	3,541	32	8,765	24,229	4,794	20
Fully vaccinated & previous infection	414	952	157	16	58	1,192	32	3
Booster vaccinated	468	1,312	306	23	528	2,873	318	11

Notes: Summary statistics are not aggregated on the primary case level. Unvaccinated includes 7 primary cases with partial vaccination and 52 contacts with partial vaccination.

Table S8: Summary statistics of vaccination status by primary case level

	Omicron				Delta			
	Primary Cases	Household Contacts	Secondary Cases	SAR (%)	Primary Cases	Household Contacts	Secondary Cases	SAR (%)
**Total**	8,568	18,038	5,229	29	18,107	42,964	8,911	21
**Immunity**								
Unvaccinated	1,166	2,936	904	31	8,611	23,694	5,086	21
Previous infection (no vaccination)	128	287	43	15	145	374	28	7
Fully vaccinated (no previous infection)	6,392	13,206	3,901	30	8,765	17,953	3,641	20
Fully vaccinated & previous infection	414	825	132	16	58	134	10	7
Booster vaccinated	468	784	249	32	528	809	146	18

Notes: This table is similar to Table S7, but with row-wise aggregation on the primary case level. For instance, there were 1,166 primary cases with Omicron that were unvaccinated. These primary cases had 2,936 household contacts of which 904 tested positive as secondary cases, implying a SAR of 31%.

Table S9: Secondary attack rate (SAR) and number of observations by vaccination status of both primary case and contact

a. Omicron households

	Contact		
**Primary case**	**Unvaccinated**	**Fully vaccinated**	**Booster vaccinated**
Unvaccinated	22% [32/146/103]	32% [150/462/300]	38% [67/176/174]
Fully vaccinated	27% [742/2,788/1,906]	30% [3,125/10,525/6,274]	21% [209/1,005/940]
Booster vaccinated	31% [381/1,237/756]	31% [493/1,568/892]	23% [30/131/119]

b. Delta households

	Contact		
**Primary case**	**Unvaccinated**	**Fully vaccinated**	**Booster vaccinated**
Unvaccinated	18% [27/154/101]	18% [72/402/291]	19% [47/253/249]
Fully vaccinated	25% [1,090/4,365/2,853]	19% [2,433/12,741/7,850]	12% [156/1,355/1,287]
Booster vaccinated	28% [2,601/9,231/5,907]	18% [2,370/13,198/6,891]	9% [115/1,265/1,136]

Notes: Numbers in each cell show: "SAR% [Number of secondary cases / Number of contacts / Number of primary cases]".

Table S10: Secondary attack rate (SAR) and number of observations by vaccination status and age, at the primary case level

a. Omicron households

	Vaccination status		
**Age**	**Unvaccinated**	**Fully vaccinated**	**Booster vaccinated**
0-10 years	36% [433/1,188/404]	25% [10/40/13]	-
10-20 years	27% [206/772/282]	18% [745/4,095/1,588]	-% [<5/16/5]
20-30 years	22% [116/525/273]	24% [942/3,900/2,366]	22% [38/171/116]
30-40 years	28% [78/279/123]	33% [732/2,210/969]	32% [56/176/94]
40-50 years	37% [41/110/49]	38% [936/2,438/976]	34% [62/183/69]
50-60 years	48% [27/56/29]	41% [562/1,362/792]	33% [29/87/53]
60-70 years	-% [<5/5/5]	54% [136/250/210]	37% [29/79/65]
70+ years	-% [<5/<5/<5]	57% [13/23/20]	44% [32/72/66]

b. Delta households

	Vaccination status		
**Age**	**Unvaccinated**	**Fully vaccinated**	**Booster vaccinated**
0-10 years	20% [2,714/13,242/4,438]	12% [13/109/37]	-
10-20 years	19% [1,163/6,073/2,126]	9% [316/3,608/1,379]	-% [<5/7/<5]
20-30 years	19% [327/1,682/845]	13% [345/2,616/1,548]	8% [5/60/39]
30-40 years	32% [478/1,509/614]	23% [727/3,095/1,230]	17% [25/143/65]
40-50 years	36% [274/765/336]	22% [1,066/4,806/1,909]	14% [22/158/67]
50-60 years	29% [90/311/170]	26% [787/2,999/1,818]	15% [18/118/68]
60-70 years	35% [33/94/65]	35% [354/1,019/854]	17% [20/117/100]
70+ years	39% [7/18/17]	34% [71/209/193]	26% [53/206/187]

Notes: Numbers in each cell show: "SAR% [Number of secondary cases / Number of contacts / Number of primary cases]".

Table S11: Secondary attack rate (SAR) and number of observations by immunity status and age, at the contact level

a. Omicron households

	Vaccination status		
Age	Unvaccinated	Fully vaccinated	Booster vaccinated
0-10 years	27% [698/2,563/1,843]	13% [18/141/121]	-
10-20 years	31% [202/644/574]	22% [629/2,846/2,192]	-% [<5/16/16]
20-30 years	21% [96/447/400]	27% [827/3,045/2,441]	21% [46/220/212]
30-40 years	30% [75/248/231]	41% [605/1,467/1,309]	32% [54/170/165]
40-50 years	35% [58/164/148]	33% [859/2,599/2,107]	23% [76/334/322]
50-60 years	26% [20/77/70]	34% [677/2,011/1,687]	22% [60/278/270]
60-70 years	29% [5/17/17]	35% [134/387/364]	22% [31/141/135]
70+ years	-% [<5/11/11]	32% [19/59/55]	24% [36/153/139]

b. Delta households

	Vaccination status		
Age	Unvaccinated	Fully vaccinated	Booster vaccinated
0-10 years	24% [1,996/8,297/6,177]	3% [8/253/228]	-
10-20 years	30% [699/2,365/2,075]	8% [495/5,873/4,538]	-% [<5/21/20]
20-30 years	28% [267/952/876]	13% [375/2,812/2,397]	-% [<5/141/137]
30-40 years	37% [405/1,094/983]	22% [1,175/5,313/4,167]	13% [91/702/663]
40-50 years	34% [225/655/595]	21% [1,565/7,347/5,709]	7% [68/924/882]
50-60 years	33% [86/260/247]	24% [821/3,436/3,033]	13% [53/403/392]
60-70 years	29% [26/91/90]	35% [371/1,066/1,031]	15% [43/282/276]
70+ years	39% [14/36/34]	27% [65/241/226]	15% [59/400/380]

Notes: Numbers in each cell show: “SAR% [Number of secondary cases / Number of contacts / Number of primary cases]”.

Table S12: Secondary attack rate (SAR) and number of observations by immunity status and household size, at the contact level

a. Omicron households

	Vaccination status		
**Household size**	**Unvaccinated**	**Fully vaccinated**	**Booster vaccinated**
2 persons	32% [103/319/319]	39% [1,020/2,606/2,606]	35% [143/414/414]
3 persons	29% [242/840/703]	29% [887/3,037/1,898]	15% [50/327/300]
4 persons	29% [520/1,766/1,109]	29% [1,298/4,443/2,064]	21% [76/361/334]
5 persons	26% [242/941/502]	24% [462/1,941/730]	19% [30/158/143]
6 persons	16% [48/305/132]	19% [101/528/168]	13% [7/52/42]

b. Delta households

	Vaccination status		
**Household size**	**Unvaccinated**	**Fully vaccinated**	**Booster vaccinated**
2 persons	37% [329/894/894]	29% [1,119/3,871/3,871]	17% [136/799/799]
3 persons	27% [595/2,180/1,710]	18% [910/5,025/3,232]	9% [47/521/483]
4 persons	28% [1,532/5,549/3,816]	18% [1,838/10,382/5,129]	8% [81/965/877]
5 persons	26% [1,013/3,869/1,937]	15% [822/5,516/2,267]	11% [49/463/409]
6 persons	20% [249/1,258/504]	12% [186/1,547/533]	4% [5/125/104]

Notes: Numbers in each cell show: "SAR% [Number of secondary cases / Number of contacts / Number of primary cases]".

4 Additional analyses

This section provides additional analyses for the robustness of our main results.

4.1 Viral load of primary cases

Figure S3 shows the density of sample Ct values of primary cases stratified by the Omicron VOC and Delta VOC.

Figure S3: Density of Ct value

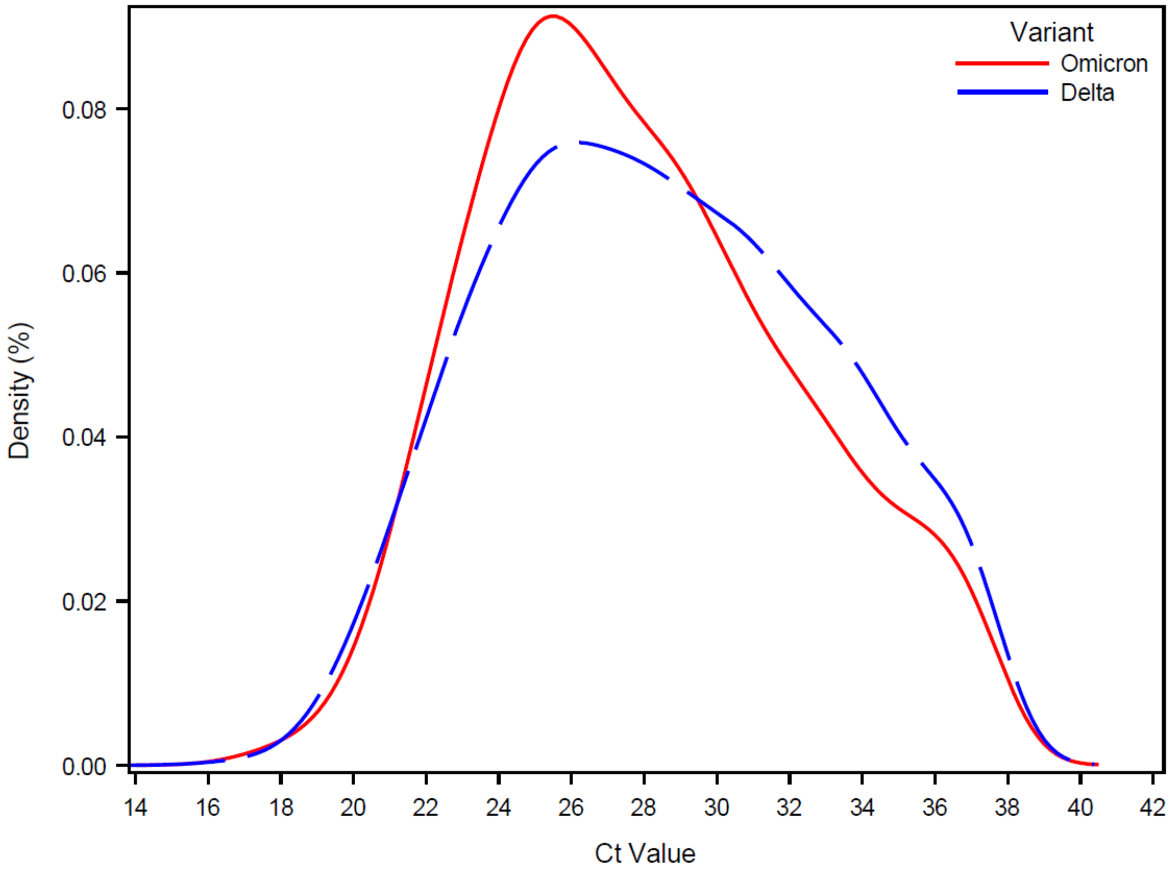

Notes: This figure shows the density of the sample Ct values of primary cases stratified by the Omicron (red) and Delta (blue) VOC.

4.2 Misclassification of cases

One of the main potential weaknesses of our approach is the assumption that primary and secondary cases are classified correctly, i.e., that the presumed within-household transmission did in fact occur from primary to secondary household cases. There are three overall concerns with misclassifications: i) Tertiary cases could be misclassified as secondary cases; ii) Misclassification of primary cases iii) Secondary cases are identified as being infected in the household, but are in fact infected by the outside community. We address these three overall concerns below. Lastly, we investigate the impact on our results from the potential pollution from misclassification of cases.

i) *Misclassification of tertiary cases as secondary cases*

Tertiary cases could in theory be misclassified as secondary cases. This should not pose an issue when comparing variants, as long as the misclassification is the same across variants. However, if one variant has a shorter serial interval time, as the Omicron VOC has been suggested to have[2], this could lead to a difference in the misclassification that is correlated with the household variant. To address this, we use two-person households as a validation measure, because they do not include tertiary cases. Figure S4, panels a-b shows the testing propensity for the overall sample (2-6 person households) as well as for 2-person households. Both the levels and trends are relatively similar across the two panels. Panel c-d show the SAR over time. Here, we see a higher level of SARs for 2-person households compared to larger households, but the trends are similar.

Figure S4: Probability of being tested and testing positive, stratified by household size

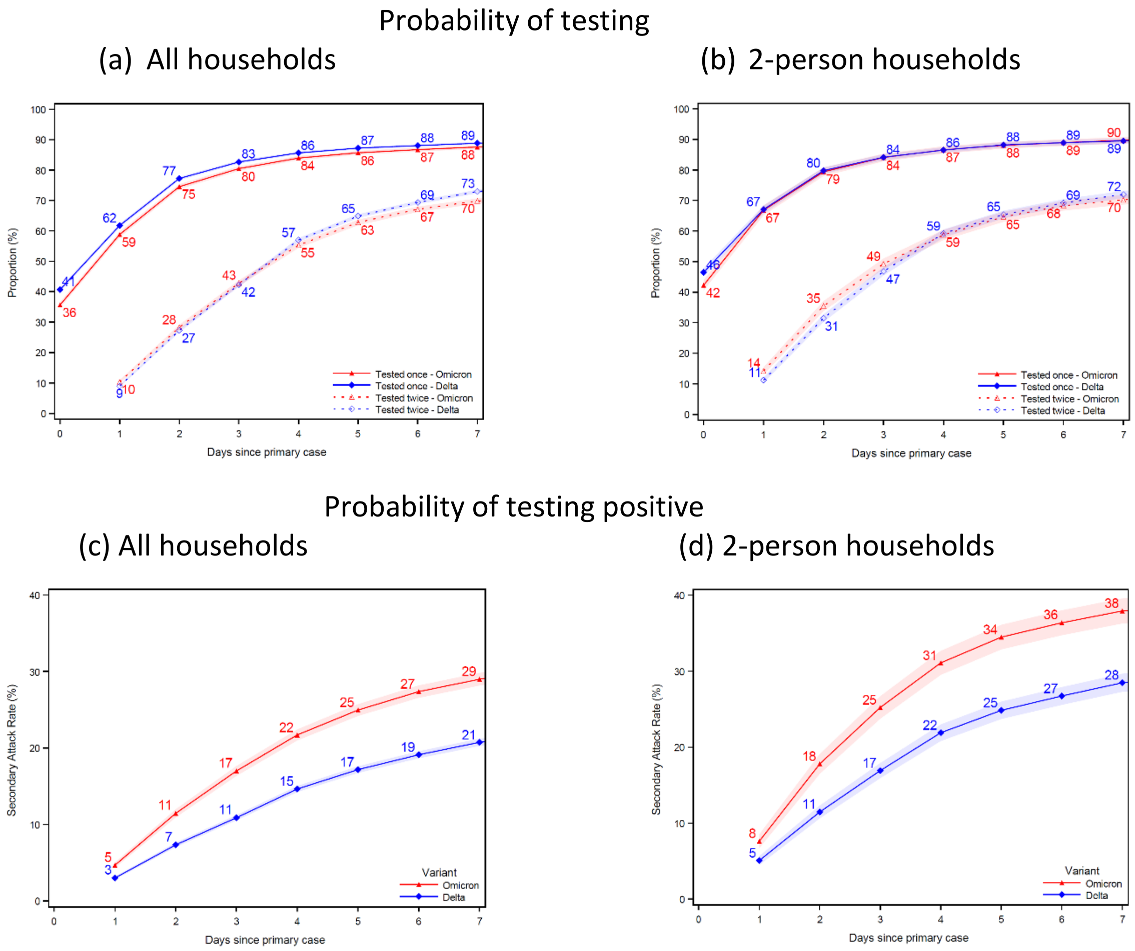

Notes: Panels a and c shows the same as Figure 1, whereas panels b and d are stratified by 2-person households. Panels a-b show the probability of potential secondary cases being tested after a primary case has been identified within the household. Panel c-d show the probability of potential secondary cases that test positive subsequently to a primary case being identified within the household. Note that the latter is not conditional on being tested, i.e., the denominator contains test negative individuals and untested individuals. The x axes show the days since the primary case tested positive, and the y axes show the proportion of individuals either being tested (a) or testing positive (b) with an RT-PCR test, based on the variant of the primary case. The SAR for each day relative to the primary case can be read directly from panels c-d. For example, in panel c the SAR on day 7 is 29% for Omicron (red) and 21% for Delta (blue), whereas the SAR on day 4 is 22% for Omicron and 15% for Delta. The markers show the point estimates of the mean. The shaded areas show the 95% confidence bands clustered on the household level.

Next, using the SAR estimates from panel b, we can calculate the relative SAR in Omicron households compared to Delta households. If the increased serial interval for Omicron implied more tertiary cases, we should see an increased difference in the relative SAR over time for households with more than two members. We find no indication of a difference in the relative SAR of households infected with the Omicron VOC relative to those infected with the Delta VOC across household size (Table

S13). This suggests that differences in the probability of misclassification of tertiary cases as secondary cases across variants is negligible, and thus not a major limitation in our study.

Lastly, we note that the levels of the SAR in two-person households differ from the SAR in larger households, which might be due to unobserved differences in characteristics related to transmission. However, we have no reason to believe any differences across variants within household size, i.e., that the two-person households infected with the Omicron VOC are inherently different from those infected with the Delta VOC.

Table S13: Relative SAR over time since primary case by household size by day since primary case

	SAR				Relative SAR	
	All households		2-person households		All households	2-person households
Day	Delta	Omicron	Delta	Omicron	(Omicron/Delta)	(Omicron/Delta)
1	3	5	5	8	1.7	1.6
2	7	11	11	18	1.6	1.6
3	11	17	17	25	1.5	1.5
4	15	22	22	31	1.5	1.4
5	17	25	25	34	1.5	1.4
6	19	27	27	36	1.4	1.3
7	21	29	28	38	1.4	1.4

Notes: The SAR estimates are presented in Figure S4 panels c-d.

ii) *Misclassification of primary cases*

Correct identification of primary cases within the household is important for this study as this determines whether the household is counted as an Omicron or Delta household when assessing the effect of the VOC on transmission. In theory the first identified case, i.e., the index case, may not be the primary case of a household transmission chain. Correct identification of primary cases is important for our estimates of infectiousness from primary cases, as infectiousness is correlated with age, vaccination status, and viral load. In our setting, we use the timing of tests and test results to classify cases. This could be an issue, if for example vaccination status and/or symptoms are correlated with the likelihood of being tested. The optimal setting would be to test all household members on, say, a daily basis to make sure of the temporal ordering between the primary and secondary cases. We do not have that, but Denmark had a high test capacity and test intensity, which leaves us with a large proportion of contacts actually having several test results within 7 days of exposure.

Overall, we can classify the relevant household contacts into five types by their observed tests and test results from two tests within 7 days of exposure:

Table S14: Classification of contacts using obtained tests and test results

Type	Test 1	Test 2	Potential primary case	Number of contacts on day 7	Number of contacts on day 14
A	None	None	Yes	7,044	5,681
B	Positive	None	Yes	4,245	4,408
C	Negative	None	No	6,384	4,779
D	Negative	Positive	No	9,895	12,338
E	Negative	Negative	No	33,434	33,796
Total				61,002	61,002

- Type A can potentially be the primary case, as we do not have any test result for them.
- Type B can potentially be the primary case, but just identified later than the index case.
- Type C, D and E cannot be the primary case, as we have a negative test result on them after exposure

We can leverage this and only include households in the analysis, where all household contacts have a negative test after the primary case, i.e., we only include households consisting of contact types C, D and E. For these households, we assume no misclassification of primary cases (assuming a high test sensitivity). This leaves us with a subsample of 72% of all households and 68% of all contacts. Using this sub-sample, we estimate our full regression model again. The estimates are relatively robust to this sub-sampling (Table S21, model XI).

Finally, to reduce the probability of misclassifying primary cases as secondary cases, we only include secondary cases found on day 2-7 and 3-7. This accounts for the possibility that an individual that was previously infected may self-present for a test the day after another person in the same household that they themselves infected. The results (Table S19, column V and VI) are qualitatively similar to the main results presented in the paper, which further supports the overall robustness of our conclusions.

iii) *Misclassification of community cases as secondary household cases*

Lastly, secondary cases could in theory be infected by the outside community and not the household and therefore be misclassified as secondary household cases. To address this potential concern of misclassification, we first investigate the probability that secondary cases are infected with the same

variant as the primary case. In households where the primary case was infected with the Omicron VOC, we found 4,090 secondary cases that also had a Variant PCR result (Table S15). Of these, 4,010 (98%) were also Omicron VOC and 80 (2%) were Delta VOC. Similarly, in households where the primary case was infected with the Delta VOC, we found 7,420 secondary cases. Of these, 7,209 (97%) were also Delta and 211 (3%) were Omicron VOC. The overall intra-household correlation of variants was 97.5 (CI: 97.1-97.8). We interpret this as the possibility of misclassification being negligible.

Table S15: Intra-household correlation of variants

I. Number of cases

	Primary case		
**Secondary Case**	**Omicron**	**Delta**	**All**
Omicron	4,010	211	4,221
Delta	80	7,209	7,289
All	4,090	7,420	11,510

II. Regression estimates

	Primary case		
	**Omicron**	**Delta**	**All**
Intra-household correlation (%)	98.0	97.2	97.5
(95%-CI)	(97.6-98.5)	(96.7-97.6)	(97.1-97.8)
Number of observations	4,090	7,420	11,510
Number of households	3,038	5,445	8,483

Notes: This table provides estimates of the intra-household correlation of variants, i.e., the probability that the primary and secondary cases are infected with the same variant. Panel I provides the number of observations. Panel II provides regression estimates. Cluster-robust standard errors are clustered on the household level.

This measure is, however, a necessary—but not sufficient—condition. If the local geographic neighborhood is primarily infected with one variant and that is the same as within the household, we would not be able to separate secondary cases infected in the household from those infected in the local community based on the variant. However, for households infected with a different variant from that which is dominant in the neighborhood, we can in fact gauge the role of misclassified community infections. To this end, we calculated the overall incidence (Figure S5, a) and the share of Omicron cases (Figure S5, b) for each of the 98 municipalities in Denmark. Thus, we can follow households infected with Omicron that are surrounded by a neighborhood with Delta. Here, we

would expect the secondary cases to be infected with Omicron, if they were infected in the household, and infected with Delta, if they were infected in the community. And vice versa for Delta households situated in Omicron neighborhoods.

Figure S5: Overall case incidence and proportion of cases with Omicron in Danish municipalities

(a) Case incidence per 100,000 inhabitants by municipality

(b) Proportion of cases with Omicron by municipality

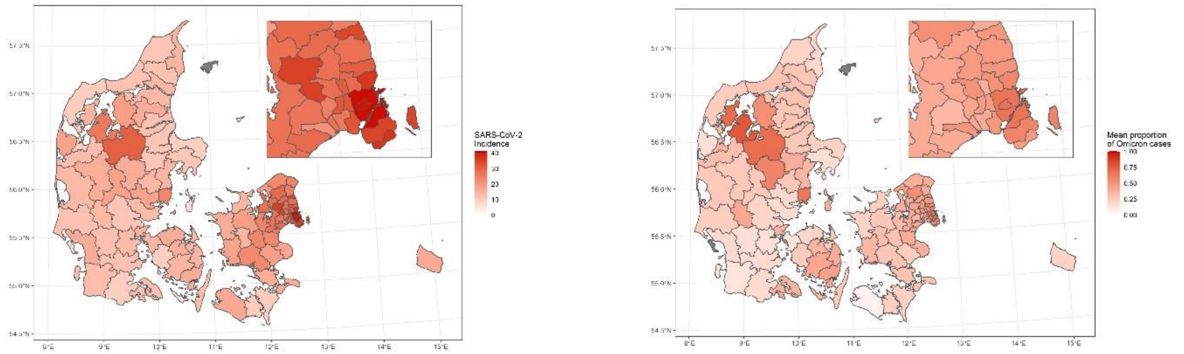

Notes: This figure shows the geospatial pattern across Danish municipalities of overall SARS-CoV-2 incidence as well as the proportion of cases with Omicron for the period 10th to 21st December 2021. Panel a shows the incidence of RT-PCR positive cases per 100,000 inhabitants. Panel b shows the proportion of positive cases with Omicron.

We categorized municipalities into four quartiles based on their proportion of Omicron cases. We then again estimated the probability that the secondary case has the same variant as the primary case. We found a strong correlation for households infected with the Omicron VOC—across all municipality incidence quartiles. For households infected with the Delta VOC, we find a 5 percentage point (on a baseline of 99%) lower probability in municipalities with the highest proportion of Omicron cases (Table S16, specification I). Moreover, we found little evidence that the estimates of misclassification were driven by households located in municipalities with either low or high overall case incidence (specification II and III). Indeed, this suggests that there is some are some contamination of household secondary cases for Delta households, but also that the misclassification is limited.

Table S16: Intra-household correlation of variants by municipality omicron case proportion

Specification	I		II		III	
Municipalities	All		Incidence > Q1		Incidence < Q4	
Primary case variant	Delta	Omicron	Delta	Omicron	Delta	Omicron
**Omicron case proportion**						
Q1 (0-25%)	99	99	99	98	99	99
Q2 (25-34%)	99	98	99	97	99	98
Q3 (34-48%)	97	98	97	98	97	99
Q4 (48-100%)	94	98	94	98	97	99
Number of observations	7,420	4,090	6,132	3,864	4,834	1,510
Number of households	5,445	3,038	4,488	2,869	3,550	1,077
Number of municipalities	97	92	70	70	72	67

Notes: The incidence quartiles are number of cases per 1,000 inhabitants: Q1=13, Q2=16, and Q3=26. The geospatial patterns of the incidence and omicron case proportions are illustrated in Figure S5.

Impact of misclassification on our estimates

If a substantial number of secondary cases were more or less randomly infected with the Delta vs the Omicron VOC from outside the household, then we expect that the within-household correlation would be lower than we observed. However, it could also be argued that a high correlation may result from a sufficiently strong local-level spatial component in the spread of variants. In this case, a natural geographical correlation in the variant with which a case is infected would be expected to affect both the primary and secondary case within the household, as the geographical location of the household is fixed. Therefore, the intra-household correlation of variants would be biased upwards compared to the real effect of secondary cases being infected by the primary case, as secondary cases are overcounted. However, the misclassification would only affect the OR estimates reported in the paper if the misclassification is not proportional to the stratum-specific odds of testing positive. If one assumes that the misclassification is proportional to the risk such that the percentage that is misclassified is the same in low- and high-risk strata. Such a proportional mechanism would work to inflate the estimates, but to a limited extent. The misclassification would also shrink the confidence intervals, but not substantially under reasonable assumptions. Appendix Table S17 shows how our effect sizes would be influenced under different levels of misclassification. We believe that the effects are unlikely to materially change the conclusions of the analyses, even under more severe assumptions that those assumed here. Table S17 shows the OR estimates with no misclassification of cases (column 1), 10% misclassification (column 2), and 30% misclassification (column 3).

Table S17: Sensitivity of OR estimates to potential misclassification of cases

	No misclassification OR	10% misclassification OR	30% misclassification OR
**Contact vaccination status**			
Delta households			
Booster vaccinated	0.41	0.42	0.43
Fully vaccinated	1.00	1.00	1.00
Unvaccinated	2.36	2.28	2.16
Omicron households			
Booster vaccinated	1.31	1.30	1.28
Fully vaccinated	2.39	2.31	2.18
Unvaccinated	2.60	2.50	2.34

Notes: This table provides hypothetical estimates of the sensitivity of the OR estimates to potential misclassification of cases using relatively extreme proportions of misclassification.

4.3 Time since vaccination for positive secondary cases

Figure S6 shows the distribution of days since last vaccination/infection for secondary cases, stratified by the household VOC and time since vaccination of both the primary and secondary case. The panels show there is no obvious trend in the waning immunity across variants. Note the groups of booster-vaccinated primary and secondary cases are from a low number of cases, which limits precision.

Figure S6: Time since vaccination for positive secondary cases

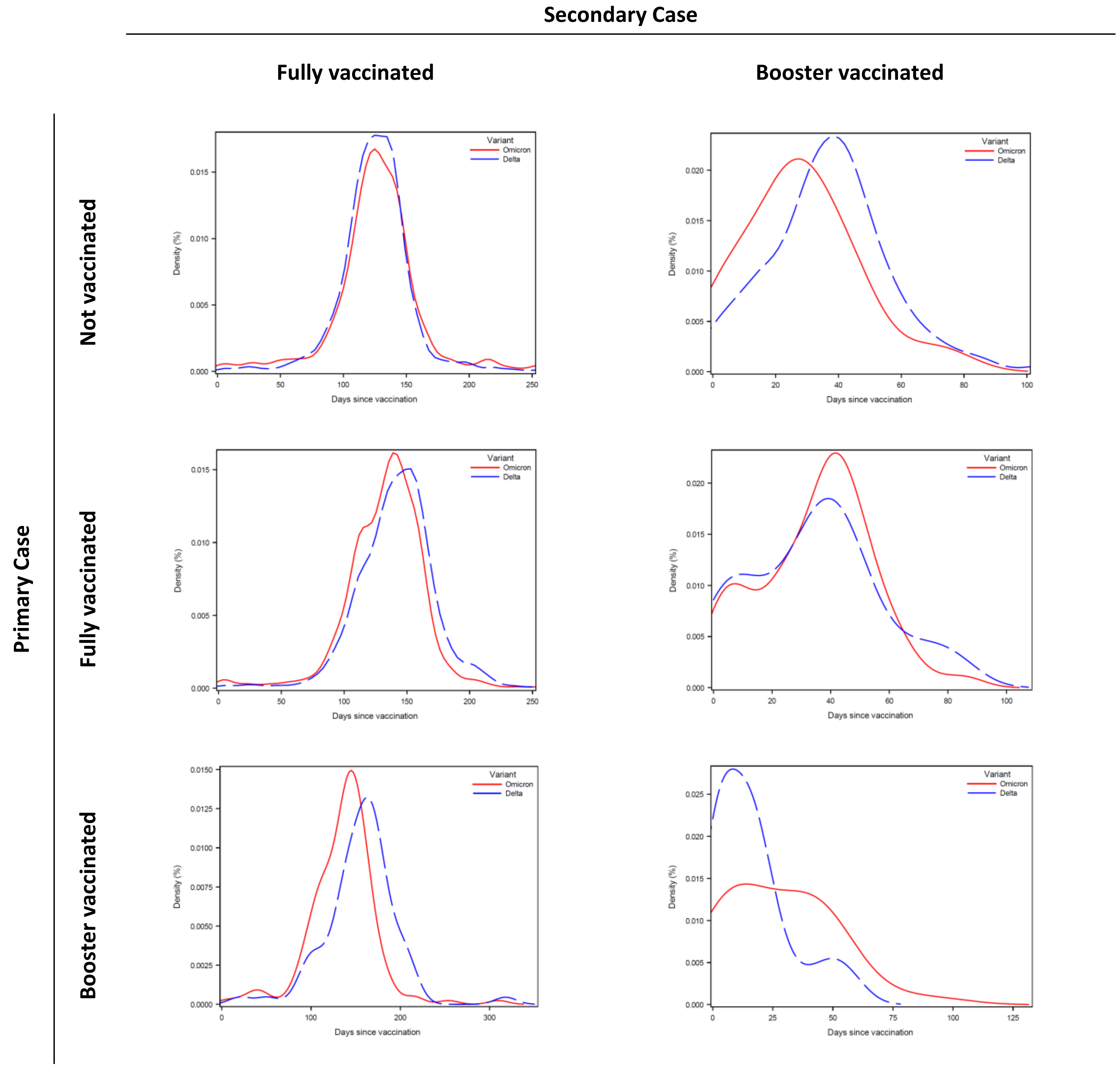

Notes: This figure shows the distribution of days since last vaccination/infection for secondary cases, stratified by the household VOC (red=Omicron, blue=Delta) and vaccination status of both the primary and secondary case.

4.4 Robustness of main results

This subsection provides results of additional analyses using different model specifications in order to validate the results shown in the main paper.

The test probabilities for RT-PCR test alone are shown in Figure S7 (this augments Figure 1, which also includes antigen tests). Similarly, Figure S8 shows the same as Figure 1, but using a 14-day follow-up period in place of the 7-day period shown in the main paper. The patterns are qualitatively similar to these different potential assumptions.

Figure S7: Probability of being tested and testing positive with an RT-PCR test

(a) Probability of testing (b) Probability of testing positive

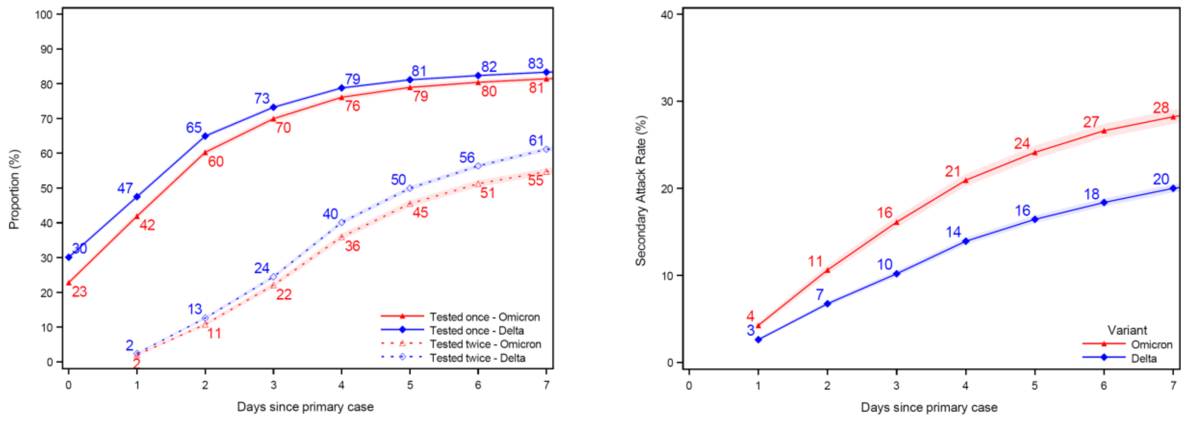

Notes: This figure shows the same as Figure 1, but only including RT-PCR tests. Panel (a) shows the probability of potential secondary cases being tested after a primary case has been identified within the household. Panel (b) shows the probability of potential secondary cases that test positive subsequently to a primary case being identified within the household. Note that the latter is not conditional on being tested, i.e., the denominator contains test negative individuals and untested individuals. The x axes show the days since the primary case tested positive, and the y axes show the proportion of individuals either being tested (panel a) or testing positive (panel b) with an RT-PCR test, based on the variant of the primary case. The SAR for each day relative to the primary case can be read directly from panel (b). For example, the SAR on day 7 is 28% for Omicron (red) and 20% for Delta (blue), whereas the SAR on day 4 is 21% for Omicron and 14% for Delta. The markers show the point estimates of the mean. The shaded areas show the 95% confidence bands clustered on the household level.

Figure S8: Probability of being tested and testing positive, 14-day follow-up

(a) Probability of testing

(b) Probability of testing positive

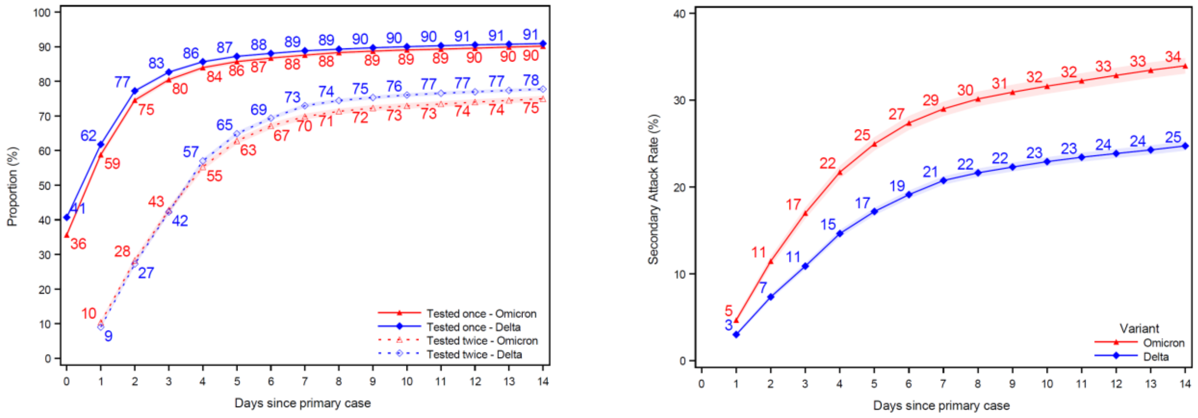

Notes: This figure shows the same as Figure 1, but with a 14-day follow-up period. Panel (a) shows the probability of potential secondary cases being tested after a primary case has been identified within the household. Panel (b) shows the probability of potential secondary cases that test positive subsequently to a primary case being identified within the household. Note that the latter is not conditional on being tested, i.e., the denominator contains test negative individuals and untested individuals. The x axes show the days since the primary case tested positive, and the y axes show the proportion of individuals either being tested (a) or testing positive (b) with an RT-PCR test, based on the variant of the primary case. The SAR for each day relative to the primary case can be read directly from panel (b). For example, the SAR on day 7 is 29% for Omicron (red) and 21% for Delta (blue), whereas the SAR on day 4 is 22% for Omicron and 15% for Delta. The markers show the point estimates of the mean. The shaded areas show the 95% confidence bands clustered on the household level.

To investigate the robustness of our main results, including the underlying assumptions, we re-ran the models to specific strata of the data. The estimates obtained from the same logistic regression model fit to each data subset are shown in Tables S18-S22, where Columns I-XII refer to the following:

I) The analysis presented in the main manuscript (for reference).

II) Using 14 days of follow-up, rather than 7 days.

III) Restricting the household contacts to those having obtained a test, rather than all members of the same household.

IV) Excluding all households with a previous infection.

V) Only including secondary cases identified on day 2-7, rather than days 1-7.

VI) Only including secondary cases identified on day 3-7, rather than days 1-7.

VII) Excluding all households with a primary case younger than 10 years.

VIII) Including only 2-person households.

IX) Excluding all individuals with partial vaccination.

X) Controlling for Ct value of the primary case using an additional explanatory variable.

XI) Only including households where all contacts have been tested negative subsequent to the primary case.

XII) Splitting the "Fully vaccinated" category into four categories for both the primary case and household contact.

The results are qualitatively similar between these 11 different analyses, which further supports the robustness of our conclusions.

In Table S23, we further provide unadjusted estimates for the infectiousness and susceptibility,

i.e., excluding the control variables age, sex, and household size.

Table S18: Robustness Analyses I

	I		II		III		IV	
	Main		14-day follow-up		Only tested contacts		No prev. HH infect.	
	**OR**	**CI**	**OR**	**CI**	**OR**	**CI**	**OR**	**CI**
**Contact vaccination status**								
Delta households								
Booster vaccinated	0.41	(0.36-0.47)	0.41	(0.37-0.46)	0.38	(0.34-0.43)	0.39	(0.35-0.45)
Fully vaccinated	ref	(.)	ref	(.)	ref	(.)	ref	(.)
Unvaccinated	2.36	(2.20-2.54)	2.24	(2.09-2.40)	2.80	(2.60-3.02)	2.13	(1.97-2.30)
Omicron households								
Booster vaccinated	1.31	(1.14-1.51)	1.27	(1.10-1.45)	1.25	(1.08-1.44)	1.35	(1.16-1.57)
Fully vaccinated	2.39	(2.24-2.54)	2.37	(2.22-2.52)	2.46	(2.31-2.62)	2.40	(2.25-2.57)
Unvaccinated	2.60	(2.35-2.87)	2.83	(2.57-3.12)	3.27	(2.93-3.64)	2.40	(2.16-2.68)
**Primary case vaccination status**								
Booster vaccinated	0.80	(0.69-0.92)	0.81	(0.70-0.94)	0.77	(0.66-0.89)	0.80	(0.69-0.94)
Fully vaccinated	ref	(.)	ref	(.)	ref	(.)	ref	(.)
Unvaccinated	1.37	(1.27-1.47)	1.40	(1.30-1.50)	1.64	(1.51-1.77)	1.39	(1.29-1.51)
**Primary case age**								
0-10	1.23	(1.11-1.36)	1.29	(1.17-1.43)	0.82	(0.74-0.92)	1.11	(0.99-1.24)
10-20	0.90	(0.82-0.99)	0.94	(0.86-1.03)	0.69	(0.63-0.77)	0.84	(0.76-0.93)
20-30	ref	(.)	ref	(.)	ref	(.)	ref	(.)
30-40	1.74	(1.58-1.92)	1.70	(1.55-1.87)	1.65	(1.48-1.83)	1.65	(1.49-1.83)
40-50	2.25	(2.04-2.49)	2.18	(1.98-2.40)	1.94	(1.75-2.16)	2.09	(1.88-2.32)
50-60	2.32	(2.10-2.57)	2.26	(2.05-2.50)	2.07	(1.86-2.31)	2.26	(2.02-2.52)
60-70	2.95	(2.53-3.44)	3.03	(2.60-3.54)	2.60	(2.20-3.07)	2.81	(2.40-3.30)
70+	4.17	(3.22-5.41)	3.94	(3.04-5.11)	3.36	(2.55-4.44)	4.04	(3.09-5.28)
**Contact age**								
0-10	0.79	(0.72-0.87)	0.81	(0.74-0.88)	0.74	(0.66-0.82)	0.82	(0.74-0.91)
10-20	0.74	(0.68-0.81)	0.75	(0.69-0.82)	0.68	(0.62-0.75)	0.74	(0.67-0.81)
20-30	ref	(.)	ref	(.)	ref	(.)	ref	(.)
30-40	1.62	(1.48-1.76)	1.58	(1.45-1.72)	1.58	(1.44-1.74)	1.60	(1.46-1.76)
40-50	1.59	(1.46-1.73)	1.48	(1.37-1.61)	1.56	(1.42-1.71)	1.53	(1.40-1.68)
50-60	1.47	(1.34-1.61)	1.39	(1.27-1.51)	1.52	(1.38-1.68)	1.42	(1.29-1.57)
60-70	1.32	(1.15-1.51)	1.21	(1.06-1.39)	1.51	(1.30-1.76)	1.31	(1.14-1.51)
70+	0.94	(0.76-1.17)	0.92	(0.75-1.14)	1.29	(1.01-1.65)	0.89	(0.71-1.11)
**Household size**								
2	2.35	(2.04-2.72)	2.03	(1.77-2.34)	1.93	(1.66-2.24)	2.20	(1.88-2.57)
3	1.53	(1.33-1.77)	1.40	(1.22-1.61)	1.36	(1.18-1.58)	1.47	(1.26-1.72)
4	1.57	(1.37-1.80)	1.49	(1.30-1.70)	1.34	(1.16-1.54)	1.52	(1.31-1.77)
5	1.40	(1.21-1.62)	1.36	(1.18-1.57)	1.24	(1.07-1.44)	1.40	(1.20-1.64)
6	ref	(.)	ref	(.)	ref	(.)	ref	(.)
**Contact sex**								
Male	ref	(.)	ref	(.)	ref	(.)	ref	(.)
Female	1.12	(1.08-1.16)	1.12	(1.08-1.16)	1.05	(1.01-1.09)	1.11	(1.07-1.16)
**Primary case sex**								
Male	ref	(.)	ref	(.)	ref	(.)	ref	(.)
Female	0.97	(0.93-1.02)	0.96	(0.92-1.01)	0.93	(0.89-0.98)	0.99	(0.94-1.04)
Number of observations	61,002		61,002		53,958		52,770	
Number of households	26,675		26,675		25,247		23,481	

Table S19: Robustness Analyses II

	I		V		VI		VII	
	Main		Only cases on day 2-7		Only cases on day 3-7		Primary cases >10years	
	**OR**	**CI**	**OR**	**CI**	**OR**	**CI**	**OR**	**CI**
**Contact vaccination status**								
Delta households								
Booster vaccinated	0.41	(0.36-0.47)	0.42	(0.36-0.48)	0.43	(0.37-0.50)	0.42	(0.36-0.49)
Fully vaccinated	ref	(.)	ref	(.)	ref	(.)	ref	(.)
Unvaccinated	2.36	(2.20-2.54)	2.26	(2.10-2.44)	2.15	(1.98-2.33)	2.32	(2.14-2.53)
Omicron households								
Booster vaccinated	1.31	(1.14-1.51)	1.25	(1.07-1.45)	1.19	(0.99-1.42)	1.29	(1.11-1.49)
Fully vaccinated	2.39	(2.24-2.54)	2.36	(2.21-2.52)	2.24	(2.08-2.41)	2.33	(2.18-2.49)
Unvaccinated	2.60	(2.35-2.87)	2.64	(2.37-2.92)	2.51	(2.24-2.82)	2.49	(2.24-2.78)
**Primary case vaccination status**								
Booster vaccinated	0.80	(0.69-0.92)	0.78	(0.67-0.91)	0.79	(0.67-0.94)	0.80	(0.69-0.92)
Fully vaccinated	ref	(.)	ref	(.)	ref	(.)	ref	(.)
Unvaccinated	1.37	(1.27-1.47)	1.38	(1.28-1.49)	1.34	(1.24-1.46)	1.36	(1.26-1.46)
**Primary case age**								
0-10	1.23	(1.11-1.36)	1.32	(1.19-1.47)	1.40	(1.25-1.58)	-	-
10-20	0.90	(0.82-0.99)	0.96	(0.87-1.06)	1.02	(0.91-1.13)	0.93	(0.84-1.02)
20-30	ref	(.)	ref	(.)	ref	(.)	ref	(.)
30-40	1.74	(1.58-1.92)	1.73	(1.56-1.92)	1.77	(1.58-1.98)	1.72	(1.56-1.90)
40-50	2.25	(2.04-2.49)	2.29	(2.07-2.54)	2.34	(2.09-2.62)	2.28	(2.06-2.51)
50-60	2.32	(2.10-2.57)	2.35	(2.11-2.61)	2.35	(2.08-2.64)	2.32	(2.10-2.57)
60-70	2.95	(2.53-3.44)	2.80	(2.38-3.30)	2.72	(2.28-3.25)	2.87	(2.46-3.36)
70+	4.17	(3.22-5.41)	4.08	(3.09-5.37)	3.87	(2.87-5.22)	4.00	(3.09-5.20)
**Contact age**								
0-10	0.79	(0.72-0.87)	0.86	(0.78-0.95)	0.95	(0.85-1.06)	0.83	(0.75-0.92)
10-20	0.74	(0.68-0.81)	0.78	(0.71-0.86)	0.82	(0.74-0.91)	0.74	(0.67-0.81)
20-30	ref	(.)	ref	(.)	ref	(.)	ref	(.)
30-40	1.62	(1.48-1.76)	1.69	(1.54-1.85)	1.77	(1.59-1.96)	1.62	(1.47-1.79)
40-50	1.59	(1.46-1.73)	1.67	(1.52-1.82)	1.71	(1.54-1.89)	1.51	(1.38-1.66)
50-60	1.47	(1.34-1.61)	1.51	(1.37-1.66)	1.54	(1.38-1.72)	1.44	(1.31-1.58)
60-70	1.32	(1.15-1.51)	1.41	(1.22-1.62)	1.45	(1.23-1.70)	1.33	(1.15-1.52)
70+	0.94	(0.76-1.17)	1.00	(0.80-1.24)	1.04	(0.81-1.33)	0.95	(0.76-1.18)
**Household size**								
2	2.35	(2.04-2.72)	2.34	(2.01-2.72)	2.21	(1.88-2.60)	2.39	(2.01-2.83)
3	1.53	(1.33-1.77)	1.52	(1.31-1.76)	1.47	(1.25-1.73)	1.50	(1.26-1.78)
4	1.57	(1.37-1.80)	1.58	(1.37-1.83)	1.57	(1.35-1.84)	1.54	(1.30-1.82)
5	1.40	(1.21-1.62)	1.42	(1.22-1.66)	1.39	(1.18-1.64)	1.33	(1.11-1.59)
6	ref	(.)	ref	(.)	ref	(.)	ref	(.)
**Contact sex**								
Male	ref	(.)	ref	(.)	ref	(.)	ref	(.)
Female	1.12	(1.08-1.16)	1.12	(1.08-1.17)	1.11	(1.07-1.17)	1.12	(1.07-1.17)
**Primary case sex**								
Male	ref	(.)	ref	(.)	ref	(.)	ref	(.)
Female	0.97	(0.93-1.02)	0.97	(0.92-1.02)	0.95	(0.90-1.00)	0.98	(0.93-1.03)
Number of observations	61,002		58,869		55,785		46,423	
Number of households	26,675		26,070		25,229		21,783	

Table S20: Robustness Analyses III

	I		VIII		IX	
	Main		2-person households		No partial vaccination	
	OR	CI	OR	CI	OR	CI
**Contact vaccination status**						
Delta households						
Booster vaccinated	0.41	(0.36-0.47)	0.37	(0.30-0.46)	0.41	(0.36-0.46)
Fully vaccinated	ref	(.)	ref	(.)	ref	(.)
Unvaccinated	2.36	(2.20-2.54)	1.94	(1.61-2.34)	2.36	(2.20-2.53)
Omicron households						
Booster vaccinated	1.31	(1.14-1.51)	1.34	(1.06-1.70)	1.31	(1.14-1.51)
Fully vaccinated	2.39	(2.24-2.54)	2.20	(1.95-2.47)	2.39	(2.24-2.54)
Unvaccinated	2.60	(2.35-2.87)	1.82	(1.40-2.37)	2.60	(2.35-2.87)
**Primary case vaccination status**						
Booster vaccinated	0.80	(0.69-0.92)	0.81	(0.66-0.99)	0.80	(0.69-0.93)
Fully vaccinated	ref	(.)	ref	(.)	ref	(.)
Unvaccinated	1.37	(1.27-1.47)	1.20	(1.03-1.40)	1.37	(1.27-1.47)
**Primary case age**						
0-10	1.23	(1.11-1.36)	0.86	(0.64-1.16)	1.23	(1.11-1.36)
10-20	0.90	(0.82-0.99)	0.70	(0.56-0.87)	0.90	(0.82-0.99)
20-30	ref	(.)	ref	(.)	ref	(.)
30-40	1.74	(1.58-1.92)	1.36	(1.12-1.65)	1.74	(1.58-1.92)
40-50	2.25	(2.04-2.49)	1.85	(1.50-2.27)	2.26	(2.05-2.49)
50-60	2.32	(2.10-2.57)	2.19	(1.83-2.62)	2.32	(2.10-2.57)
60-70	2.95	(2.53-3.44)	2.62	(2.10-3.26)	2.96	(2.54-3.46)
70+	4.17	(3.22-5.41)	3.43	(2.45-4.80)	4.17	(3.22-5.41)
**Contact age**						
0-10	0.79	(0.72-0.87)	0.65	(0.47-0.89)	0.79	(0.72-0.87)
10-20	0.74	(0.68-0.81)	0.53	(0.41-0.69)	0.74	(0.68-0.81)
20-30	ref	(.)	ref	(.)	ref	(.)
30-40	1.62	(1.48-1.76)	1.00	(0.83-1.21)	1.62	(1.48-1.77)
40-50	1.59	(1.46-1.73)	1.04	(0.84-1.28)	1.59	(1.46-1.73)
50-60	1.47	(1.34-1.61)	1.37	(1.15-1.63)	1.47	(1.34-1.60)
60-70	1.32	(1.15-1.51)	1.33	(1.07-1.65)	1.32	(1.15-1.51)
70+	0.94	(0.76-1.17)	1.01	(0.73-1.38)	0.95	(0.76-1.17)
**Household size**						
2	2.35	(2.04-2.72)	-	-	2.35	(2.04-2.72)
3	1.53	(1.33-1.77)	-	-	1.53	(1.33-1.77)
4	1.57	(1.37-1.80)	-	-	1.57	(1.37-1.80)
5	1.40	(1.21-1.62)	-	-	1.40	(1.21-1.62)
6	ref	(.)	-	-	ref	(.)
**Contact sex**						
Male	ref	(.)	ref	(.)	ref	(.)
Female	1.12	(1.08-1.16)	1.21	(1.09-1.34)	1.12	(1.08-1.16)
**Primary case sex**						
Male	ref	(.)	ref	(.)	ref	(.)
Female	0.97	(0.93-1.02)	1.05	(0.94-1.17)	0.97	(0.93-1.02)
Number of observations	61,002		8,903		60,936	
Number of households	26,675		8,903		26,663	

Table S21: Robustness Analyses IV

	I		X		XI	
	Main		Control for Ct		Only tested negative	
	**OR**	**CI**	**OR**	**CI**	**OR**	**CI**
**Contact vaccination status**						
Delta households						
Booster vaccinated	0.41	(0.36-0.47)	0.42	(0.35-0.50)	0.39	(0.33-0.45)
Fully vaccinated	ref	(.)	ref	(.)	ref	(.)
Unvaccinated	2.36	(2.20-2.54)	2.48	(2.23-2.74)	2.44	(2.21-2.69)
Omicron households						
Booster vaccinated	1.31	(1.14-1.51)	1.34	(1.08-1.67)	1.26	(1.05-1.51)
Fully vaccinated	2.39	(2.24-2.54)	2.42	(2.21-2.65)	2.39	(2.21-2.59)
Unvaccinated	2.60	(2.35-2.87)	2.96	(2.56-3.41)	3.09	(2.69-3.54)
**Primary case vaccination status**						
Booster vaccinated	0.80	(0.69-0.92)	0.74	(0.58-0.94)	0.74	(0.62-0.90)
Fully vaccinated	ref	(.)	ref	(.)	ref	(.)
Unvaccinated	1.37	(1.27-1.47)	1.29	(1.16-1.43)	1.48	(1.34-1.64)
**Primary case age**						
0-10	1.23	(1.11-1.36)	1.45	(1.25-1.68)	1.06	(0.92-1.22)
10-20	0.90	(0.82-0.99)	0.92	(0.81-1.05)	0.81	(0.71-0.92)
20-30	ref	(.)	ref	(.)	ref	(.)
30-40	1.74	(1.58-1.92)	1.63	(1.42-1.88)	1.53	(1.33-1.76)
40-50	2.25	(2.04-2.49)	2.34	(2.03-2.69)	2.11	(1.85-2.41)
50-60	2.32	(2.10-2.57)	2.31	(2.00-2.68)	2.28	(2.00-2.60)
60-70	2.95	(2.53-3.44)	2.83	(2.24-3.58)	2.48	(2.05-3.01)
70+	4.17	(3.22-5.41)	4.26	(2.87-6.30)	3.73	(2.69-5.18)
**Contact age**						
0-10	0.79	(0.72-0.87)	0.78	(0.68-0.89)	0.80	(0.70-0.92)
10-20	0.74	(0.68-0.81)	0.78	(0.69-0.89)	0.67	(0.60-0.76)
20-30	ref	(.)	ref	(.)	ref	(.)
30-40	1.62	(1.48-1.76)	1.72	(1.51-1.95)	1.56	(1.38-1.76)
40-50	1.59	(1.46-1.73)	1.67	(1.47-1.88)	1.53	(1.37-1.72)
50-60	1.47	(1.34-1.61)	1.52	(1.33-1.73)	1.46	(1.30-1.65)
60-70	1.32	(1.15-1.51)	1.46	(1.20-1.78)	1.37	(1.14-1.63)
70+	0.94	(0.76-1.17)	0.87	(0.63-1.19)	1.15	(0.86-1.54)
**Household size**						
2	2.35	(2.04-2.72)	2.47	(2.02-3.02)	2.47	(1.96-3.13)
3	1.53	(1.33-1.77)	1.57	(1.29-1.91)	1.53	(1.21-1.93)
4	1.57	(1.37-1.80)	1.51	(1.25-1.83)	1.55	(1.24-1.94)
5	1.40	(1.21-1.62)	1.47	(1.20-1.79)	1.46	(1.16-1.85)
6	ref	(.)	ref	(.)	ref	(.)
**Contact sex**						
Male	ref	(.)	ref	(.)	ref	(.)
Female	1.12	(1.08-1.16)	1.10	(1.04-1.16)	1.11	(1.06-1.17)
**Primary case sex**						
Male	ref	(.)	ref	(.)	ref	(.)
Female	0.97	(0.93-1.02)	0.98	(0.91-1.05)	0.90	(0.85-0.96)
**Primary case Ct value**						
14-16			0.83	(0.19-3.70)		
16-18			2.94	(1.80-4.80)		
18-20			1.56	(1.23-1.98)		
20-22			1.68	(1.44-1.96)		
22-24			1.32	(1.16-1.50)		
24-26			1.19	(1.05-1.35)		
26-28			1.05	(0.92-1.20)		
28-30			ref	(.)		
30-32			0.91	(0.79-1.04)		
32-34			0.89	(0.77-1.04)		
34-36			0.84	(0.71-1.00)		
36-38			0.76	(0.62-0.94)		
Number of observations	61,002		29,716		41,662	
Number of households	26,675		12,898		19,242	

Table S22: Robustness Analyses V

	XII<br>More vaccination groups	
	OR	CI
**Contact vaccination status**		
Delta households		
Booster vaccinated	0.39	(0.34-0.44)
Unvaccinated	2.12	(1.97-2.28)
Previous infection (no vaccination)	0.30	(0.22-0.41)
Fully vaccinated (no previous infection)	ref	(.)
Fully vaccinated & previous infection	0.13	(0.09-0.18)
Omicron households		
Booster vaccinated	1.25	(1.09-1.45)
Unvaccinated	2.36	(2.13-2.61)
Previous infection (no vaccination)	1.18	(0.88-1.58)
Fully vaccinated (no previous infection)	2.40	(2.25-2.56)
Fully vaccinated & previous infection	1.24	(1.03-1.50)
**Primary case vaccination status**		
Booster vaccinated	0.78	(0.67-0.90)
Unvaccinated	1.37	(1.27-1.47)
Previous infection (no vaccination)	0.61	(0.44-0.85)
Fully vaccinated (no previous infection)	ref	(.)
Fully vaccinated & previous infection	0.55	(0.45-0.68)
**Primary case age**		
0-10	1.18	(1.06-1.31)
10-20	0.87	(0.79-0.96)
20-30	ref	(.)
30-40	1.71	(1.55-1.88)
40-50	2.19	(1.98-2.41)
50-60	2.26	(2.04-2.51)
60-70	2.86	(2.45-3.34)
70+	4.08	(3.15-5.30)
**Contact age**		
0-10	0.81	(0.73-0.89)
10-20	0.73	(0.67-0.80)
20-30	ref	(.)
30-40	1.56	(1.43-1.71)
40-50	1.52	(1.39-1.65)
50-60	1.40	(1.28-1.53)
60-70	1.25	(1.09-1.43)
70+	0.89	(0.72-1.10)
**Household size**		
2	2.29	(1.98-2.65)
3	1.49	(1.30-1.72)
4	1.54	(1.34-1.77)
5	1.38	(1.20-1.60)
6		
**Contact sex**		
Male	ref	(.)
Female	1.12	(1.08-1.16)
**Primary case sex**		
Male	ref	(.)
Female	0.97	(0.92-1.02)
Number of observations	61,002	
Number of households	26,675	

Table S23: Robustness Analyses VI

	XIII		I	
	Unadjusted		Main	
	OR	CI	OR	CI
**Contact vaccination status**				
Delta households				
Booster vaccinated	0.62	(0.48-0.62)	0.41	(0.36-0.47)
Fully vaccinated	ref	(.)	ref	(.)
Unvaccinated	1.71	(1.54-1.71)	2.36	(2.20-2.54)
Omicron households				
Booster vaccinated	1.53	(1.17-1.53)	1.31	(1.14-1.51)
Fully vaccinated	2.03	(1.80-2.03)	2.39	(2.24-2.54)
Unvaccinated	1.85	(1.56-1.85)	2.60	(2.35-2.87)
**Primary case vaccination status**				
Booster vaccinated	1.30	(0.99-1.30)	0.80	(0.69-0.92)
Fully vaccinated	ref	(.)	ref	(.)
Unvaccinated	1.09	(0.98-1.09)	1.37	(1.27-1.47)
**Primary case age**				
0-10	-	-	1.23	(1.11-1.36)
10-20	-	-	0.90	(0.82-0.99)
20-30	-	-	ref	(.)
30-40	-	-	1.74	(1.58-1.92)
40-50	-	-	2.25	(2.04-2.49)
50-60	-	-	2.32	(2.10-2.57)
60-70	-	-	2.95	(2.53-3.44)
70+	-	-	4.17	(3.22-5.41)
**Contact age**				
0-10	-	-	0.79	(0.72-0.87)
10-20	-	-	0.74	(0.68-0.81)
20-30	-	-	ref	(.)
30-40	-	-	1.62	(1.48-1.76)
40-50	-	-	1.59	(1.46-1.73)
50-60	-	-	1.47	(1.34-1.61)
60-70	-	-	1.32	(1.15-1.51)
70+	-	-	0.94	(0.76-1.17)
**Household size**				
2	-	-	2.35	(2.04-2.72)
3	-	-	1.53	(1.33-1.77)
4	-	-	1.57	(1.37-1.80)
5	-	-	1.40	(1.21-1.62)
6	-	-	ref	(.)
**Contact sex**				
Male	-	-	ref	(.)
Female	-	-	1.12	(1.08-1.16)
**Primary case sex**				
Male	-	-	ref	(.)
Female	-	-	0.97	(0.93-1.02)
Number of observations	61,002		61,002	
Number of households	26,675		26,675	

Supplementary Information References

1 Stærk-Østergaard J, Kirkeby C, Christiansen LE, et al. Evaluation of diagnostic test procedures for SARS-CoV-2 using latent class models: comparison of antigen test kits and sampling for PCR testing based on Danish national data registries. *arXiv*. 2021; (published online Dec 21.) (preprint).

2 Backer Jantien A, Eggink Dirk, Andeweg Stijn P, Veldhuijzen Irene K, van Maarseveen Noortje, Vermaas Klaas, Vlaemynck Boris, Schepers Raf, van den Hof Susan, Reusken Chantal BEM, Wallinga Jacco. Shorter serial intervals in SARS-CoV-2 cases with Omicron BA.1 variant compared with Delta variant, the Netherlands, 13 to 26 December 2021. Euro Surveill. 2022;27(6):pii=2200042. https://doi.org/10.2807/1560-7917.ES.2022.27.6.2200042
