# Supplementary material for: Household transmission of the SARS-CoV-2 Omicron variant in Denmark
Source: Nat Commun. 2022 Sep 23;13:5573. doi: 10.1038/s41467-022-33328-3 (PMC9508106; doi:10.1038/s41467-022-33328-3)
Supplement: Supplementary file 3 — Reporting Summary [file 41467_2022_33328_MOESM3_ESM.pdf]

nature portfolio

Corresponding author(s): Frederik Plesner Lyngse

Last updated by author(s): Sep 7, 2022

Reporting Summary

Nature Portfolio wishes to improve the reproducibility of the work that we publish. This form provides structure for consistency and transparency in reporting. For further information on Nature Portfolio policies, see our Editorial Policies and the Editorial Policy Checklist.

Statistics

For all statistical analyses, confirm that the following items are present in the figure legend, table legend, main text, or Methods section.

n/a	Confirmed	
☐	☒	The exact sample size (*n*) for each experimental group/condition, given as a discrete number and unit of measurement
☐	☒	A statement on whether measurements were taken from distinct samples or whether the same sample was measured repeatedly
☐	☒	The statistical test(s) used AND whether they are one- or two-sided<br>*Only common tests should be described solely by name; describe more complex techniques in the Methods section.*
☐	☒	A description of all covariates tested
☐	☒	A description of any assumptions or corrections, such as tests of normality and adjustment for multiple comparisons
☐	☒	A full description of the statistical parameters including central tendency (e.g. means) or other basic estimates (e.g. regression coefficient) AND variation (e.g. standard deviation) or associated estimates of uncertainty (e.g. confidence intervals)
☐	☒	For null hypothesis testing, the test statistic (e.g. *F*, *t*, *r*) with confidence intervals, effect sizes, degrees of freedom and *P* value noted<br>*Give P values as exact values whenever suitable.*
☒	☐	For Bayesian analysis, information on the choice of priors and Markov chain Monte Carlo settings
☒	☐	For hierarchical and complex designs, identification of the appropriate level for tests and full reporting of outcomes
☒	☐	Estimates of effect sizes (e.g. Cohen's *d*, Pearson's *r*), indicating how they were calculated

Our web collection on statistics for biologists contains articles on many of the points above.

Software and code

Policy information about availability of computer code

Data collection	We performed no data collection or sequencing specifically for this study.
Data analysis	We used SAS 9.4 to manage and analyze the data. The code used for this study can be downloaded from a public repository: https://github.com/Flyngse/SARS-CoV-2_OmicronDelta_HouseholdTransmission

For manuscripts utilizing custom algorithms or software that are central to the research but not yet described in published literature, software must be made available to editors and reviewers. We strongly encourage code deposition in a community repository (e.g. GitHub). See the Nature Portfolio guidelines for submitting code & software for further information.

Data

Policy information about availability of data

All manuscripts must include a data availability statement. This statement should provide the following information, where applicable:

- Accession codes, unique identifiers, or web links for publicly available datasets
- A description of any restrictions on data availability
- For clinical datasets or third party data, please ensure that the statement adheres to our policy

The data used in this study are available under restricted access due to Danish data protection legislation. The data are available for research upon request to The Danish Health Data Authority and Statens Serum Institut and within the framework of the Danish data protection legislation and any required permission from Authorities. We performed no data collection or sequencing specifically for this study.

Field-specific reporting

Please select the one below that is the best fit for your research. If you are not sure, read the appropriate sections before making your selection.

☒ Life sciences ☐ Behavioural & social sciences ☐ Ecological, evolutionary & environmental sciences

For a reference copy of the document with all sections, see nature.com/documents/nr-reporting-summary-flat.pdf

Life sciences study design

All studies must disclose on these points even when the disclosure is negative.

Sample size	No predetermined sample size calculation was performed. Our data include the full population of Denmark.<br>The first Danish case infected with the Omicron VOC was detected on 22nd November 2021, and community transmission was present by early December 2021. On 8th December, Danish authorities discontinued intensive contact tracing of close contacts for cases specifically infected with the Omicron VOC. We therefore chose a study period beginning on 9th December 2021, when cases of both variants were treated approximately equally, thus reducing bias from the earlier intensified contact tracing and active case finding of the Omicron VOC. The end of the inclusion period for primary cases was set to 15th December, with household contacts followed up to 7 days after the primary case, i.e., until 22nd December 2021. We chose this as the end of our study period because Christmas holidays in Denmark often start on 23rd December, and often include extended family visits, which interrupts the typical transmission pattern within households.<br>These restrictions leaves us with a sample of 26,675 primary cases (8,568 with the Omicron VOC) and 14,140 secondary infections among 61,002 household contacts, which should be a sufficient large sample for the purpose of our study.
Data exclusions	We exclude households with less than one and more than six members. We excluded households with a positive RT-PCR test 60 days prior of the primary case and households where the primary case was ambiguous because two individuals tested positive on the same day.
Replication	The code used for this study can be downloaded from a public repository: https://github.com/Flyngse/SARS-CoV-2_OmicronDelta_HouseholdTransmission. The analysis is performed on administrative register data. The analysis can be replicated with data access through the Statens Serum Institut and Danish Health Data Authority.
Randomization	Randomization was not applicable, as the study uses observational data from administrative registers.
Blinding	Blinding was not applicable, as the study uses observational data from administrative registers.

Reporting for specific materials, systems and methods

We require information from authors about some types of materials, experimental systems and methods used in many studies. Here, indicate whether each material, system or method listed is relevant to your study. If you are not sure if a list item applies to your research, read the appropriate section before selecting a response.

Materials & experimental systems

n/a	Involved in the study	
☒	☐	Antibodies
☒	☐	Eukaryotic cell lines
☒	☐	Palaeontology and archaeology
☒	☐	Animals and other organisms
☒	☐	Human research participants
☒	☐	Clinical data
☒	☐	Dual use research of concern

Methods

n/a	Involved in the study	
☒	☐	ChIP-seq
☒	☐	Flow cytometry
☒	☐	MRI-based neuroimaging
